# Assessment of cleaning methods on bacterial burden of hospital privacy curtains: a pilot randomized controlled trial

**DOI:** 10.1038/s41598-021-01198-2

**Published:** 2021-11-08

**Authors:** Kianna Cadogan, Sabrin Bashar, Saul Magnusson, Rakesh Patidar, John Embil, Justin P. Gawaziuk, Monika Gawthrop, Song Liu, Ayush Kumar, Sarvesh Logsetty

**Affiliations:** 1 College of Medicine, BSc Med Research Program, Max Rady College of Medicine, Rady Faculty of Health Sciences, University of Manitoba, Winnipeg, MB Canada; 2 Department of Microbiology, University of Manitoba, Winnipeg, MB Canada; 3 Manitoba Firefighters’ Burn Unit, Health Sciences Centre, Winnipeg, MB Canada; 4 Section of Infectious Diseases, Department of Medicine, Max Rady College of Medicine, Rady Faculty of Health Sciences, University of Manitoba, Winnipeg, MB Canada; 5 Department of Biosystems Engineering, Faculty of Agricultural and Food Sciences, University of Manitoba, Winnipeg, MB Canada; 6 Departments of Surgery, Psychiatry and Children’s Health, Max Rady College of Medicine, Rady Faculty of Health Sciences, University of Manitoba, GF431-820 Sherbrook Street, Winnipeg, MB R3A 1R9 Canada

**Keywords:** Bacteria, Clinical microbiology

## Abstract

Healthcare-associated infections (HAIs) are an important global issue, leading to poor patient outcomes. A potential route of transmission of HAIs is through contact with hospital privacy curtains. The aim of this study is to evaluate cleaning on reduction of curtain bacterial burden. In this pilot cluster randomized controlled trial we compared the bacterial burden between three groups of 24 curtains on a regional burn/plastic surgery ward. A control group was not cleaned. Two groups were cleaned at 3–4 day intervals with either disinfectant spray or wipe. The primary outcome was the difference in mean CFU/cm^2^ between day 0 to day 21. The secondary outcome was the proportion of curtains contaminated with Methicillin-resistant *Staphylococcus aureus* (MRSA). By day 21, the control group was statistically higher (2.2 CFU/cm^2^) than spray (1.3 CFU/cm^2^) or wipe (1.5 CFU/cm^2^) (*p* < 0.05). After each cleaning at 3–4 day intervals, the bacterial burden on the curtains reduced to near day 0 levels; however, the level increased again over the intervening 3–4 days. By day 21, 64% of control curtains were contaminated with MRSA compared to 10% (spray) and 5% (wipe) (*p* < 0.05). This study show that curtains start clean and progressively become contaminated with bacteria. Regularly cleaning curtains with disinfectant spray or wipes reduces bacterial burden and MRSA contamination.

## Introduction

Healthcare-associated infections (HAIs) are responsible for significant health system and individual-level burden, including increased length of stay and exposure to antimicrobial resistant organisms such as Methicillin-resistant *Staphyloccus aureus* (MRSA)^1–4^. Annually in Canada, over 220,000 hospitalized individuals contract HAIs (7.9% of inpatients^3^), resulting in morbidity and related mortality. An estimated 8000 Canadians die every year from HAI^5^. While it is clear from published reports that benefits of reducing HAIs would outweigh the costs, data is lacking on how best to reduce potential routes of transmission. Better understanding of the sources and routes of transmission is essential to developing effective prevention strategies which can reduce this significant burden in Canada and worldwide.

Hospital environments play an important part in HAI transmission^6^. Hospital privacy curtains are an important site of environmental contamination for several reasons. First, curtains are frequently touched by patients, visitors and healthcare workers. This touching may lead to these contamination with bacteria, including antibiotic-resistant organisms (AROs)^7,8^ such as MRSA. A cross-sectional study evaluating the rate of contamination of curtains on a burns/plastic surgery ward by our group identified MRSA on 31% of the privacy curtains^7^. A subsequent longitudinal study demonstrated that the number of bacteria present on the curtains increased over time and most curtains were contaminated with MRSA 14 days after being placed in the health care environment^8^. Like many bacteria, in the absence of adequate environmental cleaning, MRSA can survive and stay viable on environmental surfaces for up to seven months^9^. Curtains are also an important site of environmental contamination as healthcare workers may be less likely to wash or disinfect their hands after contact with inanimate objects, such as curtains, as compared to after direct patient contact^10^. As such, pathogens causing HAIs may be transmitted to patients through contact with these high-touch surfaces^9,11,12^. While methods such as handwashing and cleaning high touch surfaces are strategies to reduce HAI transmission, these practices are not consistently implemented. Handwashing compliance among care providers is not 100%, and high-touch surfaces are frequently missed or not routinely cleaned^12^. While the Public Health Association of Canada’s recommendations for reducing HAIs includes integrated cleaning and monitoring of patient environments, such guidelines currently do not exist in acute-care facilities for cleaning curtains. Curtains require significant resources to remove and clean. There is little research to guide cleaning processes or frequency. At our institution changing of the curtains only occurs when curtains appear visibly soiled or if the patient has known MRSA. Prior studies have identified that hospital curtains are a potential source of HAI, however examining effective cleaning practices that are easily implemented has not been investigated. This information is needed to provide preliminary evidence to fuel both HAI prevention programs and facilitate healthcare organization policy regarding clean and safe environments for patient care. Such policy is essential to providing safe patient care environments, especially in the current context of awareness of environmental spread of pathogens. To address this need, we conducted a longitudinal, cluster randomized controlled trial to compare HAI bacterial burden over a 3-week period in a Canadian burns/plastic surgery ward. The goal of this pilot study is to determine if cleaning hospital privacy curtains with 2 cleaning methods (a hydrogen peroxide disinfectant spray, or hydrogen peroxide disinfectant wipe) reduces the overall bacterial burden, including MRSA, on hospital privacy curtains over a 3-week period. For this study, hydrogen peroxide cleaning products were used because of their known efficacy on soft environmental surfaces^13^ and their availability as commercial products^6^. The cleaning agents and processes are approved; however, the comparative efficacy of the cleaning methods is not known. The primary aim of this study is to determine the effect of cleaning on bacterial burden on curtains over time. A secondary aim is to determine the proportion of curtains contaminated with MRSA.

## Methods

### Sampling strategy, cleaning methodology, and microbial contamination evaluation

**Figure 1:**
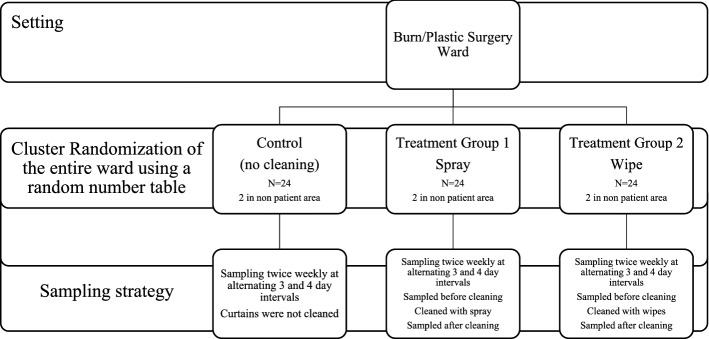
Methodology scheme.

This study was conducted on a burns/plastic surgery ward in a Canadian regional burn centre and was approved by the University of Manitoba Human Research Ethics Board. The methodology scheme is shown in Fig. 1.

In this pilot cluster randomized controlled trial we compared HAI bacterial burden between two treatment groups of polyester-blend privacy curtains (cleaned at regular intervals) and one control group which was not regularly cleaned (standard practice). Treatment groups were cleaned with 2 commercially available products: (treatment 1) hydrogen peroxide 1.4% disinfectant spray (spray) or (treatment 2) 1.4% disinfectant wipe (wipe) (Clorox, Brampton, Ontario). Twenty-four curtains were a pragmatic sample size determined by the number of beds on the ward. An additional two curtains were hung in a non-patient area to evaluate the bacterial burden on a curtain that was not touched.

#### Sampling

Cultures were collected from 26 polyester blend privacy curtains over a 3-week period. On day 0 for each group, 24 freshly commercially laundered standard hospital privacy curtains were hung one per patient bed in double (n = 8) and four-person (n = 2) occupancy patient rooms (2 additional curtains were hung in non-patient rooms). Curtains were sampled at alternating 3–4 days schedules, for a total of 24 cultures for each curtain. Cluster randomization of the entire ward as a cluster, was undertaken for the entire ward for each arm to minimize the potential confounding effect of having potentially high and low bacterial burden curtains adjacent to each other. The order of the groups (treatment 1-spray, treatment 2-wipe, control/no treatment) was assigned randomly using a random number table. At our institution, curtains are not routinely cleaned on a set schedule, but are removed and changed if visibly soiled or upon discharge if a patient is identified as having an ARO (e.g., MRSA). To avoid introducing potential bias in the decision to change the curtains, the healthcare and custodial staff not involved in the research were blinded to treatment/control group. Instead of cutting the curtain and washing the fabric in saline to elute all possible bacteria, a non-destructive sampling method was used as we wanted to evaluate the longitudinal effect of the cleaning strategy over time. As well, because the curtains were ‘privacy curtains, hanging around patient beds, it would not be appropriate to have holes in them.

#### Treatment groups (spray, wipe)

**Figure 2:**
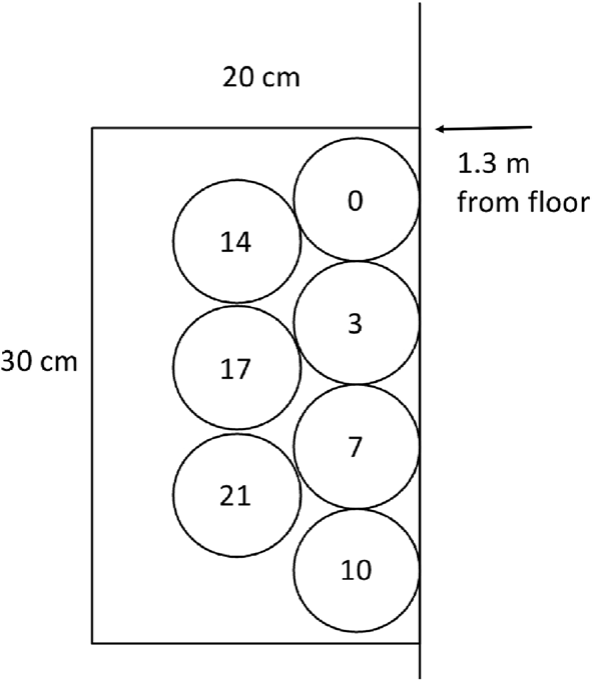
Location of curtain sampling by study day. The circles indicate the location of contact plate sampling on each day, to minimize the potential effect of residual agar left on the curtain from a prior day’s sampling.

Two samples (per time point) were taken from the treatment groups: the curtains were sampled, cleaned and then sampled again after the cleaning. Sampling was done twice weekly on alternating 3- and 4-day intervals. Each subsequent sampling day, the sample was taken from a new area of the marked out 20 cm × 30 cm space (Fig. 2) to reduce any effect from residual agar. For the purposes of the study, this was the only area cleaned on the curtain. In the spray group, the curtains were cleaned by applying five sprays on each side of the outlined section of the curtain. The spray was applied and the curtain was not further manipulated; there was no friction/wiping. The amount of spray was chosen as it allowed the surface of the curtain to be wetted by the agent, but the solution was contained and did not drip. In the wipe group, the curtains were cleaned using two passes of the wipes with one wipe simultaneously pressed against each side of the outlined section of the curtain.

Samples were obtained by contacting the agar of the plate to each side of the curtain for 30 s. A second sampling of the same region as the pre-treatment contact was taken at least three minutes after cleaning to allow time for the cleaning agent to be effective.

#### Control group (no cleaning)

One sample was obtained per time point. Sampling was done twice weekly on alternating 3- and 4-day intervals. Each subsequent sampling day, the sample was taken from a new area of the marked out 20 cm × 30 cm on the edge of the curtain and 1.3 m from the ground to reduce any effect from residual agar. This region was chosen as observations of caregivers in previous studies identified this as the highest touch area^8^ (Fig. 2).

#### Contact sampling and procedure

For this study, we used Dey/Englay Neutralizing Agar Rodac Contact Plates (#RE111103, Oxoid, Canada). Contact plates were gently pressed onto curtain surfaces to acquire samples. Curtain contact plates were incubated at 37 °C for 48 h. Colony forming units (CFUs) were counted from each plate. For the phenotypic identification of MRSA, bacterial colonies were streaked onto the mannitol salt agar (MSA) (with 6 μg/mL oxacillin; Oxoid, Canada) and incubated at 37 °C for 48 h. As a reference strain for the presence of MRSA, SA003 (CA-MRSA #40065) was used. All MSA-oxacillin-positive isolates were further genotypically confirmed by nuclease (*nuc*) and *mecA* by the colony PCR method using the protocol and primers described previously^7^.

#### Cleaning staff training and study documentation

All cleaning was done by staff trained in the protocol. The training sessions were provided by the research team and lasted approximately 10 min. Cleaning the curtains at this sample site was relatively easy for our custodial staff. Staff learned the technique quickly and provided feedback on the cleaning protocol which was incorporated into the study. On the sampling days, the number and location of curtains removed due to standard hospital procedure were recorded. MRSA colonization status of all patients was also recorded anonymously. The duration of the study was 21 days based on our previous longitudinal study that demonstrated a significant bacterial burden and MRSA contamination occurred by this time^8^.

### Statistical analysis

The primary outcome of interest in this study was the mean increase in bacterial burden (CFU/cm^2^) between day 0 and the last day the curtain was present. To assess increases in mean bacterial burden we used a paired t-test if data were normally distributed or a Wilcoxon U-test for non-parametric data. The secondary outcome was the proportion of curtains contaminated with MRSA between treatment and control groups. The MRSA rate was compared using a Fisher’s Exact test. The R programming language was used for analysis^14^. Groups were considered statistically different when *p* < 0.05 (two-tailed).

## Results

**Figure 3:**
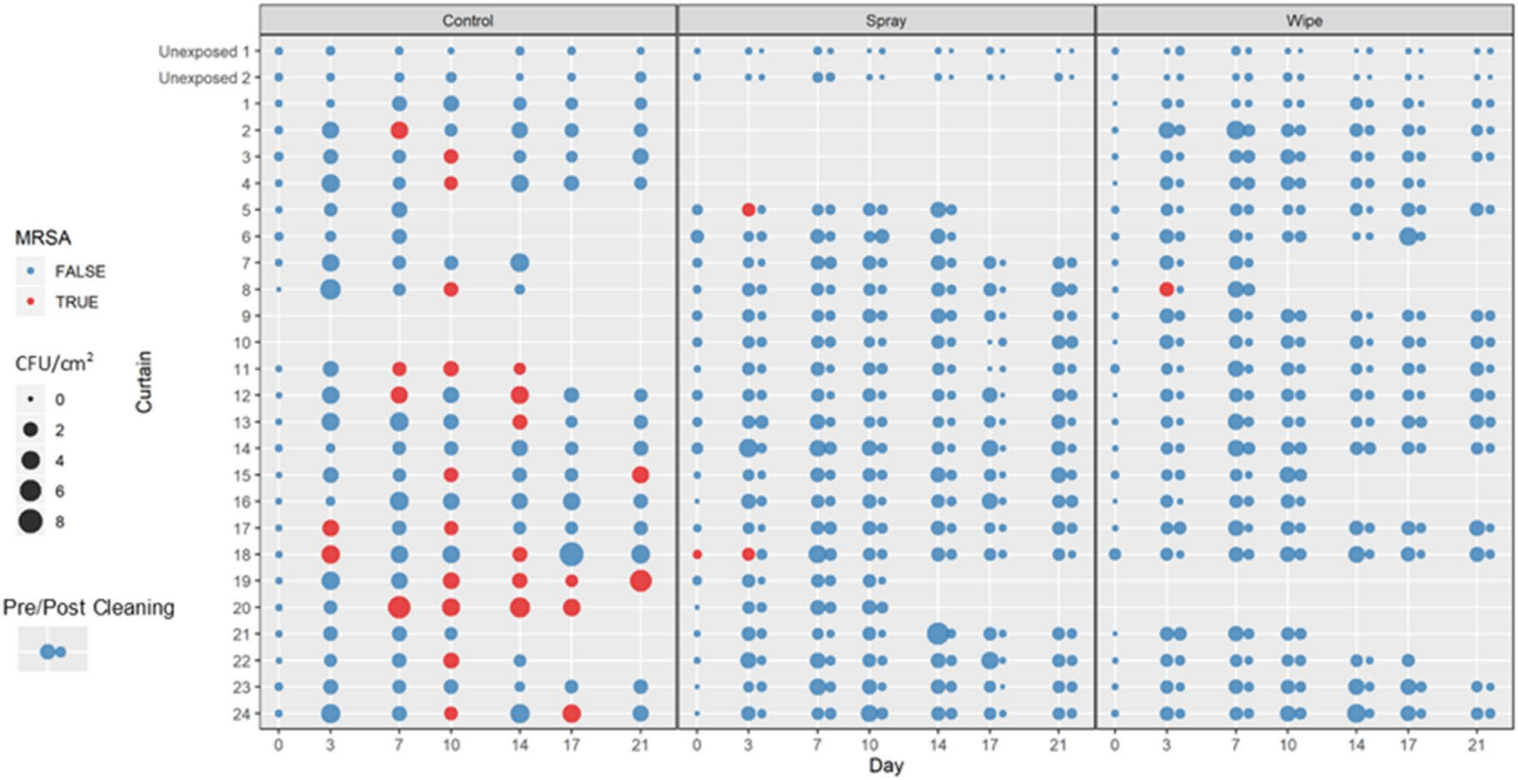
Pre/Post cleaning CFU/cm^2^ and presence of Methicillin-resistant *Staphylococcus aureus* (MRSA). Data shown as CFU/cm^2^ at each time point. The size of the circles indicates the degree of contamination with a larger circle being more contaminated. MRSA colonization on the curtain is shown with a red circle.

**Figure 4:**
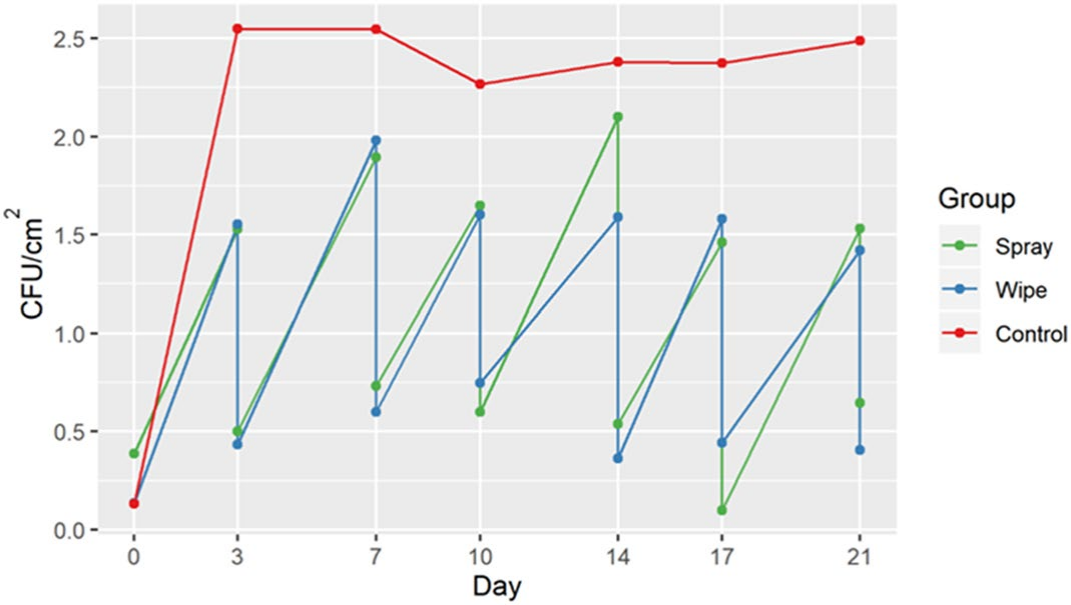
Pre/Post cleaning CFU/cm^2^.

Overall, 687 samples were obtained from 64 curtains over a 3-week period (not including the six curtains in nonpatient areas). While 24 curtains were hung, due to logistical challenges such as curtains being removed within a day of being hung, the number of curtains evaluated was 22 in the control group, 20 in the spray group and 22 in the wipe group. Overall, curtains were hung for a mean of 18 days (range 7–21). In the control group, there were 376 total curtain days (curtains × days), compared to 384 in treatment group 1 (spray) and 389 in the treatment group two (wipe). We noted no change in appearance of the curtains over time due to application of hydrogen peroxide or discoloration due to contact with the agar plate. There was no significant difference in the proportion of MRSA positive patients occupying the beds between all three groups. There was also no difference in mean bacteria whether a curtain was in a 2-person or 4-person room. In all three groups, untouched curtains (hung in a non-patient area) had minimal increase in CFU/cm^2^ (< 0.48 CFU/cm^2^) at day 21 and no MRSA contamination. There was no significant difference in the initial CFU/cm^2^ on day 0 between the curtain groups. However, the CFU/cm^2^ by the last day the curtains were hanging was significantly higher in the control compared to the spray or wipe groups: 2.2 CFU/cm^2^ in the control group, 1.3 CFU/cm^2^ in the spray group (*p* = 0.004) and 1.5 CFU/cm^2^ in the wipe group (*p* = 0.04) (Table 1). By using the cleaning methods investigated in this study, we found that it was possible to reduce the bacterial burden on curtains, including MRSA (Fig. 3). There was no significant difference between the spray and wipe groups. In both cleaning groups, cleaning reduced CFU/cm^2^ to near day 0 levels (*p* < 0.05) (Fig. 4). The bacterial burden again increases over time indicating that regular cleaning is necessary. In both cleaning groups bacterial CFU/cm^2^ after a 4-day interval was higher than after a 3-day interval (Table 2). With respect to presence of MRSA on the curtains, we found that by day 21, 64% (14/22) of curtains in the control group had been contaminated with MRSA compared to 10% (2/20) in treatment group 1, and 5% (1/22) in treatment group two (*p* < 0.05). Overall, there were also significantly more individual samples that yielded MRSA in the control group than either treatment group (Table 3).

**Table 1:** Change in CFU/cm^2^ from day 0 to last day hung.

Group	N	Days Hung; mean, (range)	Precleaning CFU/cm^2^ on last day hung; mean (SD)	CFU/cm^2^ difference (last day hung—day 0); mean (SD)	*p *value for CFU/cm^2^ (compared to control)
Control	22	18 (7–21)	2.34 (1.32)	2.21 (1.31)	–
Spray	20	19 (10–21)	1.65 (0.52)	1.26 (0.52)	0.004
Wipe	22	18 (7–21)	1.66 (0.80)	1.53 (0.78)	0.04

**Table 2:** CFU/cm^2^ 3 days versus 4 days since last cleaning.

Cleaning delay	N	CFU/cm^2^, mean ± SD
3 day	115	1.6 ± 1.2
4 day	107	1.8 ± 1.2
*p *value	–	0.04

**Table 3:** Methicillin-resistant *Staphylococcus aureus* (MRSA) contaminated samples.

Group	N	MRSA positive samples N (%)	*P *value against control*
Control	150	27 (18%)	–
Spray	266	3 (1.1%)	< 0.0004
Wipe	271	1 (0.4%)	< 0.0001

*There was no statistical difference between spray and wipe groups.

## Discussion

In this study, we demonstrated that hospital privacy curtains not only become contaminated with bacteria, including MRSA, over a relatively short period, but also that hydrogen peroxide cleaning methods significantly reduced the presence of bacteria. While this effect is encouraging, it does appear to be limited in that cleaning needs to occur at set intervals due to the increase in bacterial growth found in this study. Despite the increase in bacterial growth after cleaning, frequent cleaning may reduce certain bacterial growth over time. Interestingly, after the first cleaning, there were no MRSA colonies recovered from the cleaned curtains despite an increase in overall CFU/cm^2^. This suggests that although there is not a persistent effect on overall bacterial burden, there may be such a long-lasting effect on MRSA; this finding requires further investigation. This study addresses the gap in the literature, demonstrating that it is possible to reduce the bacterial burden on curtains using commercially available products.

One of the challenges in determining the best protocol for management of bacterial burden on curtains is the lack of guidance as to what the threshold for bacterial burden should be. To the best of our knowledge, there are no clear standards for acceptable levels of bacterial burden on hospital surfaces. The standard for food preparation equipment is < 2.5 CFU/cm^2^; it has been proposed that hospitals should be at least as clean as food preparation environments^15^. To explore this, we evaluated the number of samples over 2.5 CFU/cm^2^ (Table 4). Cleaning the curtains at a set interval appears to reduce the proportion of curtains above this threshold. In the control group 35% of samples were above 2.5 CFU/cm^2^, compared to 11% in the spray (*p* < 0.0001) and 12% in the wipe group (*p* < 0.0001). Research has identified hospital curtains as a potential route of transmission in HAI outbreaks^11^; our findings support these findings and demonstrates curtain contamination occurs shortly after clean curtains are placed in hospital rooms. While handwashing is an important form of HAI prevention, it may be that hospital curtains intercept the potential benefit. At our institution, staff hand hygiene audits have found greater than 85% compliance on this ward. Observation for these audits is conducted through a number of organizational groups, using both overt and covert observation protocols and guided by principles provided by the World Health Organization, Public Health Agency of Canada, and Infection Prevention and Control Canada.

**Table 4:** Curtains above 2.5 CFU/cm^2^.

Group	N	Curtains above 2.5 CFU/cm^2^	*p *value (against control)*
Control	124	35%	–
Spray (before)	122	11%	< 0.0001
Wipe (before)	124	12%	< 0.0001

*There was no statistical difference between spray and wipe groups.

## Limitations and strengths

This study had several limitations. First, some of the curtains were lost during the study due to regular hospital protocols for removing curtains, reducing our sample size. Additionally, although we recorded patients that were flagged for MRSA throughout the treatment groups, our institutional Infection Prevention and Control protocols do not mandate universal screening for MRSA and therefore we cannot conclusively report on the distribution of MRSA colonized patients throughout the study. Additionally, there was not sufficient information to determine source of MRSA (patient, visitor, healthcare provider). While the results of this study indicate that cleaning with a spray or wipe is an effective way to reduce pathogen contamination in the hospital environment, the role of bacteria on curtains directly causing HAIs has yet to be determined. Future studies are required to evaluate if reducing the bacterial burden on curtains leads to decreased pathogen transmission to patients and therefore decreased HAIs.

A strength of this study is that the patients, and care team were blinded to the presence and type of cleaning reducing potential bias through the cluster randomization. Additionally, two methods of cleaning were evaluated, and both found to be effective, giving options for generalizability. A further strength is the inclusion of curtains in non-patient areas to confirm that the increase in bacteria did not occur in the absence of people contacting them.

## Future directions

This study demonstrates that it is possible to reduce the bacterial burden on privacy curtains using the tested products. However, it is not known if there is any corresponding reduction in adverse clinical outcomes. Further work is needed to evaluate if a regular cleaning protocol can result in the reduction of bacterial cross-transmission in a clinically meaningful manner, such as reducing HAIs (e.g., urinary tract, or central venous catheter infections), as well as the effect on biofilms. Future randomized controlled trials will be useful to evaluate if cleaning curtains on a regular schedule influences HAIs or transmission of AROs.

While the current study focused on bacterial burden, the current COVID-19 pandemic has highlighted the importance of viral contaminants in the environment. Data suggests that SARS-CoV-2 may persist in the environment for up to 3 days^16^. Hydrogen peroxide is effective not only on bacterial cells and endospores but also viruses including SARS-CoV-2^17–19^. Moreover, hydrogen peroxide is approved by Health Canada and the United States Food and Drug Administration as a hard surface disinfectant effective against SARS-CoV-2^20^. Thus, future work should also evaluate the effect of cleaning curtains on the presence of viral pathogens.

## Conclusions

The results of this study show that hospital privacy curtains start clean and progressively become contaminated with bacteria, including MRSA. This study demonstrates that cleaning curtains with hydrogen peroxide spray or wipes significantly reduces bacterial burden and minimizes the presence of AROs. As regular cleaning was required to maintain this effect, results of this study suggest that curtains should be cleaned at least every 3 days. Information gained from this study provides preliminary evidence that can be used to fuel both HAI prevention programs and facilitate healthcare organization policy regarding clean and safe environments for patient care.
